# Immune phenotypes predict survival in patients with glioblastoma multiforme

**DOI:** 10.1186/s13045-016-0272-3

**Published:** 2016-09-01

**Authors:** Haouraa Mostafa, Andrej Pala, Josef Högel, Michal Hlavac, Elvira Dietrich, M. Andrew Westhoff, Lisa Nonnenmacher, Timo Burster, Michael Georgieff, C. Rainer Wirtz, E. Marion Schneider

**Affiliations:** 1 Sektion Experimentelle Anaesthesiologie, University Hospital Ulm, Albert Einstein Allee 23, 89081 Ulm, Germany; 2 Klinik für Anaesthesiologie, University Hospital Ulm, Albert Einstein Allee 23, 89081 Ulm, Germany; 3 Department of Neurosurgery, University Hospital Ulm Albert Einstein Allee 23, 89081 Ulm and Bezirkskrankenhaus Günzburg, Ludwig-Heilmeyer-Str. 2, 89312 Günzburg, Germany; 4 Institute for Human Genetics, Albert Einstein Allee 11, 89081 Ulm, Germany; 5 Department of Pediatric Hematology and Oncology, University Hospital Ulm, Prittwitzstr. 43, 89075 Ulm, Germany

**Keywords:** Glioblastoma multiforme, CD8+ lymphocytes, NK cells, CD39-ectonucleotidase, Recursive partitioning analysis, Survival

## Abstract

**Background:**

Glioblastoma multiforme (GBM), a common primary malignant brain tumor, rarely disseminates beyond the central nervous system and has a very bad prognosis. The current study aimed at the analysis of immunological control in individual patients with GBM.

**Methods:**

Immune phenotypes and plasma biomarkers of GBM patients were determined at the time of diagnosis using flow cytometry and ELISA, respectively.

**Results:**

Using descriptive statistics, we found that immune anomalies were distinct in individual patients. Defined marker profiles proved highly relevant for survival. A remarkable relation between activated NK cells and improved survival in GBM patients was in contrast to increased CD39 and IL-10 in patients with a detrimental course and very short survival. Recursive partitioning analysis (RPA) and Cox proportional hazards models substantiated the relevance of absolute numbers of CD8 cells and low numbers of CD39 cells for better survival.

**Conclusions:**

Defined alterations of the immune system may guide the course of disease in patients with GBM and may be prognostically valuable for longitudinal studies or can be applied for immune intervention.

**Electronic supplementary material:**

The online version of this article (doi:10.1186/s13045-016-0272-3) contains supplementary material, which is available to authorized users.

## Background

Glioblastoma multiforme (GBM) is the most common primary brain tumor in adults. Despite intensive research efforts and a multimodal management that consists of surgery, radiotherapy, and chemotherapy with temozolomide, the prognosis is very poor [1]. The hypoxic environment represents a very potent stimulus for angiogenesis [2], while molecular alterations, cell survival mechanisms, the acquisition of chemo- and radioresistance [3], and tumor heterogeneity [4] as well as multiple immune escape mechanisms are further important factors that contribute to the inferior prognosis in GBM [5, 6]. In addition to age, a number of stress factors, including chemical exposure, and demographical aspects influence the incidence of tumor manifestation [7]. At least one study demonstrated an increased risk for glioblastoma by alcohol consumption [8]. When compared with other malignancies, modulation of immune suppressor effector cells appears to be of major importance [9], and individual tumor patients may require an individual treatment regimen against immune suppressive elements. We addressed here immune phenotypes in patients with glioblastoma at the time of diagnosis. We applied flow cytometry and validated enzyme-linked immunosorbent assay (ELISA) as a reliable technique to systematically analyze immune cells in peripheral blood as well as cytokines. In general, immune modulation initiated by the tumor may require direct cell-cell contact or may occur via soluble factors, including interleukins and chemokines. At present, malignancies manifesting distantly from the peripheral immune system have not been systematically described to influence immune phenotypes. However, an important work published by Kmiecik and colleagues [10], which was performed in a small group of GBM patients, was highly promising in implying the sensitization of immune cells against GBM. In addition, the work published by Skog and coworkers [11] contributed to our understanding of effector pathways induced by the interaction of tumor-derived microparticles with target cells. Cytokines, including interleukin (IL)-10 and ferritin, secreted by tumor-infiltrating immune cells and by the tumor itself are members of a larger scenario of a tumor-associated proteome [12]. They may play an important role in downregulating major histocompatibility complex (MHC)-class I and natural killer (NK)-ligands [13]. Here, we asked whether immune phenotypes of patients with manifest GBM display distinct features differing from healthy individuals. In addition to the abovementioned IL-10 and ferritin associated with GBM, we found that the relative amounts of activated NK cells in addition to T cell receptor (TCR) α/β-positive and CD8-positive lymphocytes display a strong correlation with increased survival in GBM in bivariate analysis. When using multivariable Cox proportional hazards model analysis, the Karnofsky Performance status Scale (KPS), the isocitrate dehydrogenase-1 (IDH-1) mutation status, and high CD8 and low CD39 cells are most relevant for better survival.

## Methods

### Patients

Patients had been diagnosed for glioblastoma multiforme grade IV according to the criteria of the World Health Organization (WHO). Immune competence was tested at the time of diagnosis before surgery following standard cluster of differentiation (CD) and activation markers for T-, NK-, monocyte-, and granulocyte subpopulations according to international guidelines for whole blood flow cytometric analysis. Immune phenotyping and inflammatory and anti-inflammatory biomarkers performed by a validated ELISA was an essential step in classifying patients for their immune competence. These tests were done for each patient as part of routine care. Patients were then under high dose steroid treatment. All patients’ data were pseudonymized by a data bank held in the Section of Experimental Anaesthesiology (http://www.uniklinik-ulm.de/struktur/kliniken/sektionen/klinik-fuer-anaesthesiologie/experimentelleanaesthesiologie/home/general-information/address-contact.html). The study was conducted after approval by the local Ethics Committee of the University Hospital Ulm with the approval umber 162/10 named “Novel experimental approaches in brain tumors”. The universal trial number is U111-1179-3127. Brain tumor material was used to establish cell lines. Two milliliters of ethylenediaminetetra-acetic acid (EDTA) anti-coagulated blood were routinely analyzed from all patients at the time of diagnosis before surgery. A total of 51 patients were assessed. The mean age was 56.8 years (range 22–78 years). All patients underwent surgical resection, radiotherapy, and chemotherapy and had routine follow-up with magnetic resonance imaging interpreted by neuroradiologists. All patients are listed in Table 1.

**Table 1:** Patients’ list showing age and sex, tumor location, total or subtotal resection, O^6^-methylguanine-DNA methyltransferase (MGMT), IDH-1 mutation status, and overall survival

Patient no.	Age/sex	Tumor location	Resection	MGMT	IDH mutation	Overall survival (months)
#1	60/m	Ttemporo-occipital	Subtotal	*p*	*n.a.*	31
#2	62/m	Temporal	Total	*n*	*n*	9
#3	41/f	Fronto-parietal	Total	*n*	*n*	18+
#4	65/m	Frontal	Total	*p*	*n*	10
#5	34/m	Frontal	Subtotal	*p*	*n*	19+
#6	34/f	Occipital	Total	*n*	*n.a.*	*n.a.*
#7	44/f	Frontal	Subtotal	*n*	*n*	11
#8	54/m	Temporal	Total	*n*	*n*	23
#9	66/m	Parietal	Subtotal	*n*	*n*	25
#10	29/m	Temporal	Total	*p*	*p*	43+
#11	68/m	Parieto-occipital	Total	*n*	*n*	14
#12	71/m	Temporo-occipital	Subtotal	*n*	*n*	16+
#13	78/f	Frontal	Biopsy	*p*	*n*	15
#14	70/m	Temporal	Subtotal	*n*	*n*	10+
#15	45/m	Parieto-occipital	Subtotal	*p*	*p*	12+
#16	61/m	Temporo-occipital	Subtotal	*n*	*n*	11+
#17	52/m	Temporal	Total	*n*	*n*	10+
#18	63/f	Frontal	Total	*n*	*n*	7
#19	61/f	Frontal	Total	*p*	*n.a.*	53+
#20	41/m	Fronto-parietal	Subtotal	*n*	*n*	33
#21	75/m	Frontal	Subtotal	*n*	*n*	3
#22	74/f	Temporal	Subtotal	*p*	*p*	52
#23	57/m	Temporal	Subtotal	*n*	*n*	23
#24	22/m	Frontal	Subtotal	*p*	*n*	77
#25	56/m	Fronto-parietal	Subtotal	*n*	*p*	7
#26	44/m	Parietal	Subtotal	*p*	*p*	18+
#27	51/m	Frontal	Total	*p*	*n*	10
#28	67/f	Frontal	Total	*n.a.*	*p*	28+
#29	53/f	Frontal	Total	*n*	*n*	20+
#30	45/m	Temporo-parietal	Subtotal	*p*	*p*	31
#31	58/m	Frontal	Total	*n*	*n*	9+
#32	58/m	Frontal	Total	*n*	*p*	7+
#33	53/f	Temporo-occipital	Subtotal	*p*	*n*	5
#34	67/f	Frontal	Total	*p*	*n*	8+
#35	62/m	Frontal + temporal	Total	*n*	*n*	8+
#36	67/m	Temporal	Total	*n*	*n*	9+
#37	74/m	Occipital	Subtotal	*n*	*n*	9+
#38	54/m	Temporal	Subtotal	*n.a.*	*n*	7
#39	70/m	Frontal	Total	*p*	*n*	5+
#40	40/m	Temporo-occipital	Subtotal	*p*	*n*	61+
#41	68/f	Temporo-occipital	Total	*p*	*n*	6
#42	38/m	Parietal	Total	*p*	*n*	5+
#43	76/f	Temporal	Near total	*p*	*p*	25+
#44	72/f	Parietal	Subtotal	*n*	*p*	*n.a.*
#45	53/f	Temporal	Total	*n*	*n*	8+
#46	39/m	Frontal	Total	*p*	*p*	120+
#47	77/f	Pareital	Total	*p*	*n*	7+
#48	75/f	Temporo-parietal	Subtotal	*p*	*p*	4+
#49	24/f	Parietal	Total	*n*	*p*	85+
#50	64/m	Temporo-parietal	Subtotal	*p*	*n*	4+
#51	65/f	Fronto-parietal	Near total	*n*	*n*	4+

All patients labeled with “+” remained to be alive until to date

*n* negative, *p* positive, *m* male, *f* female, *n.a.* not available

### Healthy donors

A total of 36 healthy volunteers were included as controls. Their mean age was 36.1 years (range 19–68 years). Their immune phenotypes and plasma biomarkers were also based on routine analysis in the context of routine clinical examination. Results of all healthy donors were also pseudonymized before processing for statistical analysis.

### Flow cytometry

Surface markers of whole blood leukocytes were determined by standard flow cytometric analyses using FACScalibur and Cellquest software (BD Biosciences.com). Leukocytes were gated into lymphocytes, monocytes, and granulocytes by forward- and side-scatter analysis. Quantification of the percent-positive cells was determined by the binding of fluorescently labeled antibodies and determination of expression densities of individual antigens was recorded. The expression density of the relevant antigens was calculated as the mean fluorescence intensity (MFI) using the following formula: (% positives × mean expression density of the relevant antigen) − (% positives × mean expression density of the respective isotype control). The following antibodies were determined on lymphocytes: IgG1 and IgG2a isotype control antibodies, monoclonal antibodies directed against CD3ε-chain (clone UCHT1, Beckmancoulter.com), TCRα/β (clone WT31), CD56 (clone My31), CD4 (clone SK3), CD25 (clone M-A251), CD8 (clone 2ST8.5H7, Beckman-Coulter.com), CD16 (NK-P15), CD95 (clone DX-2), CD127 (clone #40131, RnD Systems), and CD39 (clone A1, abdserotec.com), all purchased from BD Biosciences.com, if not stated otherwise. Since all patients were analyzed during high steroid treatment, we also addressed the phenotype of two GBM patients before and after steroid medication (Additional file 1: Figure S1).

### Plasma biomarkers

Plasma from patients’ blood and control subjects was used for the quantification of biomarkers. A highly sensitive and validated ELISA (Immulite 1000®, Siemens.com) was used. The following Immulite 1000® kits were used: I10 to quantify IL-10 and FER to quantify plasma ferritin.

### MGMT analysis and IDH-1 mutation status

To analyze MGMT-promoter methylation, DNA was extracted from snap-frozen tumor tissue. A pyrosequencing platform (ID or Q24 instrument, Qiagen.com) was applied for the quantification of methylation status at a single base solution. Quantitative methylation values within a sequence context were determined for individual or multiple CpG sites. (http://www.varionostic.de/services/dna-methylation/)

IDH-1 mutation status was tested by a monoclonal antibody directed against the most frequent mutation site: IDH1 R132H [14].

### Statistics

Descriptive statistics, including medians, interquartile ranges, and 10^th^ and 90^th^ percentiles were applied to characterize leukocyte blood parameters and surface antigens in healthy donors vs. controls. Between-group comparisons of continuous variables were performed using the Mann-Whitney *U* test. The Kaplan-Meier method and log-rank test were applied to summarize and compare the overall survival and time to progression between trials or different conditions using GraphPadPrism version 6.0 or the SAS procedure “LIFETEST”. In this context, median values were used as cutoffs to dichotomize continuous traits for survival analysis. For the purpose of prediction, Cox proportional hazards models were fitted using SAS PROC PHREG. Only parameters which showed a *p* value < 0.2 in univariable analysis (Additional file 2: Table S1) were included in the multivariable Cox proportional hazards model (Additional file 3: Table S2). Unbiased recursive partitioning analysis (RPA) was applied to identify the risk classes. Absolute numbers of leukocytes and leukocyte subpopulations were determined by routine Coulter counting determinations. For all statistical investigations, tests for significance were two-tailed, with a *p* value threshold of 0.05 indicating “statistical significance”.

## Results

### IL-10 and plasma ferritin are elevated in GBM

**Fig. 1:**
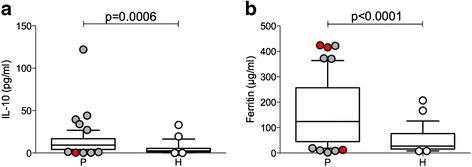
Increased median value of IL-10 and ferritin in plasma samples of GBM patients. Interleukin 10 (IL-10) (**a**), plasma ferritin (**b**) as relevant biomarkers determined in plasma samples of GBM patients (*P*) and healthy controls (*H*). Median values for IL-10 is 7.18 (±1.2 pg/ml) in elderly healthy donors, and ferritin serum concentrations may range a lot in elderly. IDH-1 mutated patients outside of the 25–75 boxed percentile are shown as *red-filled circles*

We determined two biomarkers in the patients’ plasma before surgery: IL-10 and plasma ferritin. The median values of IL-10 and ferritin were significantly elevated in the GBM patients (Fig. 1). Patients with IDH-1 mutations are labeled in red.

### CD3 expression densities are reduced in GBM

**Fig. 2:**
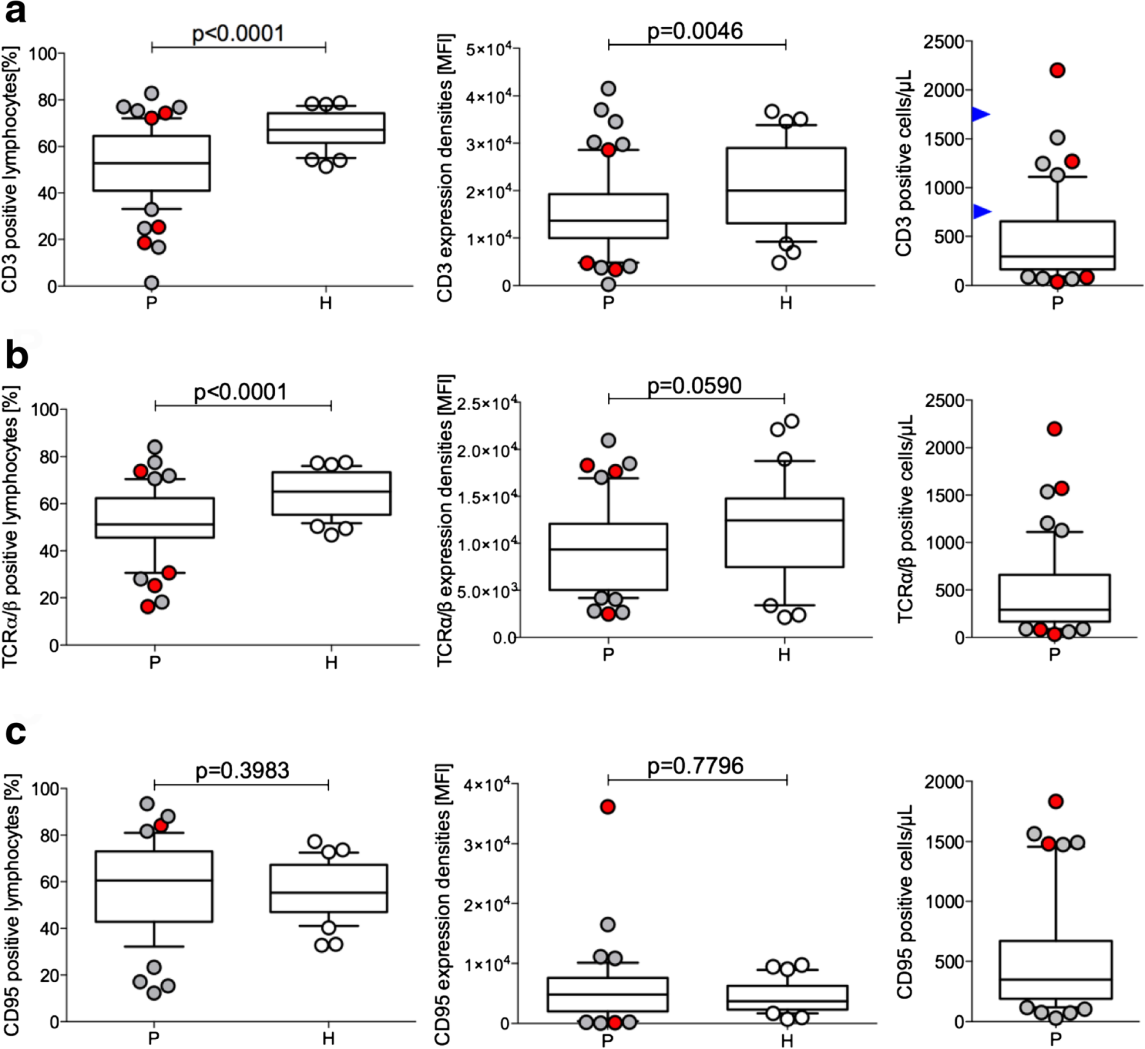
Significantly decreased CD3 and TCR α/β in peripheral blood cells but no significant change in CD95. Percent CD3 lymphocytes in EDTA whole-blood-derived lymphocytes of GBM patients (*P*) and controls (*H*) (**a**, *left*) and the corresponding CD3 expression densities (mean fluorescence intensity (MFI)) (**a**, *middle*) and absolute numbers of CD3-positive lymphocytes in patients (**a**, *right*). Normal ranges of absolute numbers are indicated as *blue arrowheads* on the *y*-axis (**a**, *right*). Percent TCRα/β lymphocytes in GBM patients and healthy controls (**b**, *left*) and the corresponding MFI distribution (**b**, *middle*) and absolute numbers of TCRα/β cells/μl blood (**b**, *right*). Percent CD95 lymphocytes in GBM patients as compared to controls (**c**, *left*) and the corresponding distribution of CD95 MFI (**c**, *middle*) and absolute CD95-expressing lymphocytes (**c**, *right*). IDH-1 mutated patients outside of the 25–75 boxed percentile are shown as *red-filled circles*. IDH-1 mutated patients outside of the 25–75 boxed percentile are shown as *red-filled circles*

In standard flow cytometry to test differentiation antigens (defined by CD clusters) in peripheral blood cells of patients with GBM, several markers displayed different profiles compared to healthy donors. In particular, we found a reduced relative amount of CD3-positive lymphocytes in the lymphocyte gate of GBM patients (*p* < 0.0001). In addition, the mean expression density of CD3 as determined by the MFI value was also significantly decreased (*p* = 0.0046) when compared with profiles obtained from healthy donors (Fig. 2a). Ranges of reference numbers of absolute CD3-positive cells are indicated as blue arrowheads. Furthermore, the relative amount for the TCRα/β determined by the WT31 clone was also significantly diminished (*p* < 0.0001; Fig. 2b). Most remarkable was the large variation in CD3- and TCRα/β-positive lymphocytes in the GBM patients when compared with the healthy controls. However, when plotting the MFI values, both the GBM patients and healthy controls displayed a larger expression range (CD3 MFI: GBM: 261–41,500; controls: 4,800–36,700 and TCRα/β: GBM: 2,400–21,000; controls: 2,100–23,000), which indicates that a difference between GBM and the controls for immune competence (CD3, TCRα/β) is likely to be due to receptor expression densities and the presence of other lymphocyte subpopulations in GBM which are not observed in healthy donors. To determine whether these alterations were potentially related to lymphocyte activation states, the amount and expression densities of CD95 were also plotted. The lymphocytes of GBM patients expressed slightly more CD95 than those of healthy donors (Fig. 2c). One GBM patient had a very high CD95 MFI (#22); however, absolute numbers of CD95-positive lymphocytes are highly elevated in five patients, two of which are IDH-1 mutated. These two patients (#32, #15) also had elevated CD3 and TCRα/β-positive lymphocyte counts.

### NK cell subpopulations in GBM and healthy donors

**Fig. 3:**
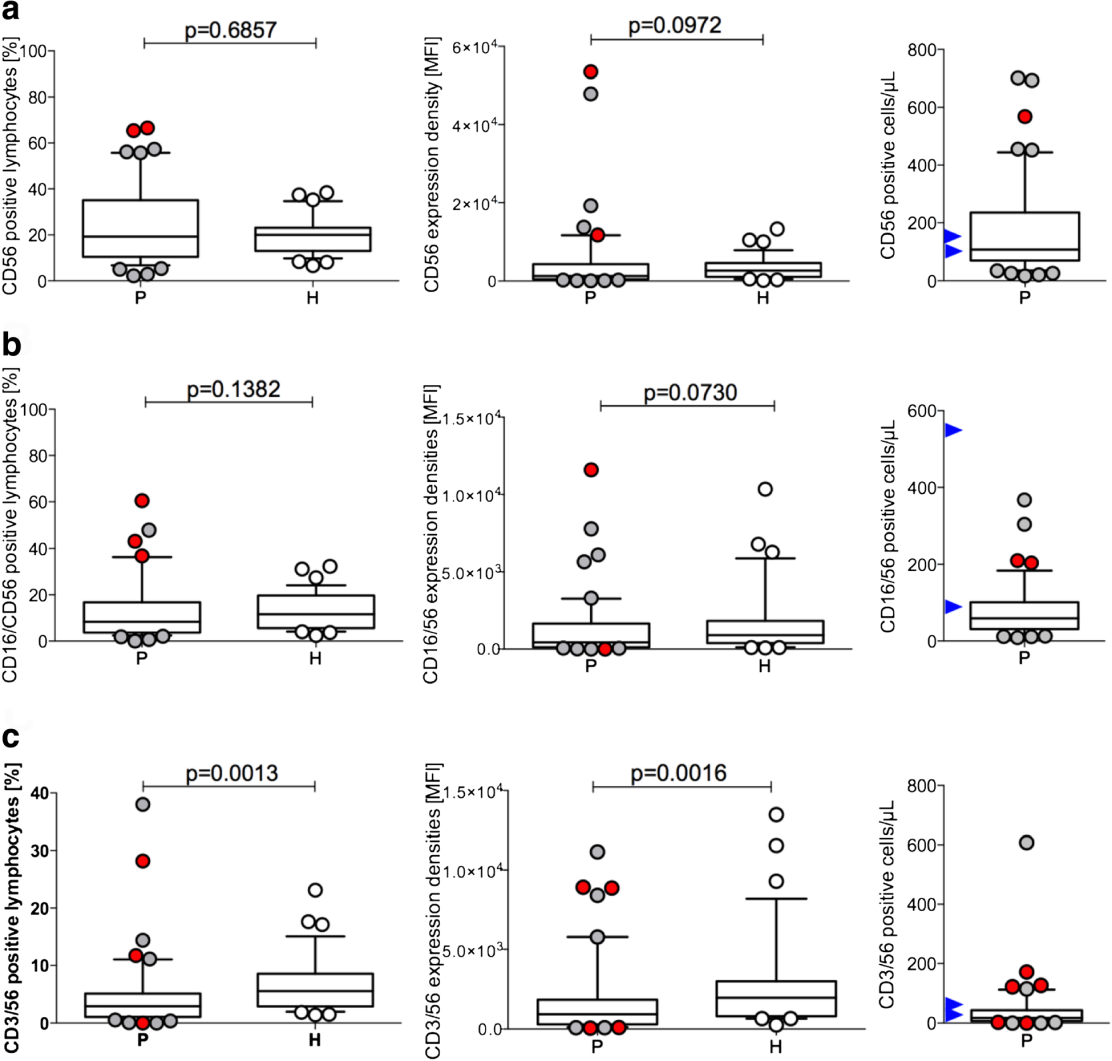
Differences in CD56 and CD16/56 NK cells and CD3/56 cytokine-activated NK (CIK) cells in GBM patients and controls. Percent CD cluster-positive lymphocytes are shown for patients (*P*) and healthy donors (*H*) on the *left*; relative expression densities, given as MFI are shown in the *middle column*, and absolute numbers of CD cluster-positive lymphocytes for the patients are shown on the *right* half of the figure. CD56 NK cells (**a**). CD56/16 co-expressing, activated NK cells (**b**). CD3/56 co-expressing, cytokine-induced killer (CIK) cells (**c**). IDH-1mutated patients outside of the 25-75 boxed percentile are shown as *red-filled circles*

Similar data sets are displayed in Fig. 2; the relative and absolute amounts of CD56^+^ NK-cells are presented in Fig. 3. Although the percentage of CD56+ NK cells varied with a large range in GBM, the median values were not significantly different from healthy donors (*p* = 0.6857; Fig. 3a). However, when the MFI values were calculated, there was a trend for lower CD56 expression in GBM when compared with the lymphocyte fraction of healthy donors (*p* = 0.0972; Fig. 3a). Absolute numbers of CD56^+^ NK-cells again demonstrate that some patients are unique and differ from values determined in healthy donors of advanced age [15]. Generally, these results suggest that GBM patients may have more CD56^dim^ NK cells than cytokine-producing CD56^bright^ cells. We further investigated CD56+ NK cells co-expressing the activation antigen CD16. The majority of GBM samples did not differ from the control (Fig. 3b). However, there were unique individual GBM patients with very high relative amounts of CD16-positive NK cells, indicating NK-cell activation. When plotting MFI values, individuals with a high CD16 expression in CD56-positive NK cells could also be identified (Fig. 3b). Nevertheless, of particular interest were patients #12, #21, #23, and #26, who displayed elevated or very elevated CD16/CD56-positive NK cells before surgery; however, only #6 had high amounts of absolute CD16/CD56-positive lymphocytes, all within the normal range of healthy individuals at an advanced age [15]. NK cells co-expressing CD3, a T-cell receptor structure, in addition to NK markers define a cell type which is particularly sensitive to cytokine activation and have been termed cytokine-induced killer (CIK) cells; they exhibit non-MHC-restricted cytolysis activities against tumor cells [16]. Such CD3/56 co-expressing effectors were clearly diminished in the majority of GBM patients (Fig. 3c, *p* = 0.0016), but two patients (#14 and #25) were characterized by a very high proportion of this cell type. An attenuated phenotype was also detected in a few healthy donors. The absolute numbers of CIK were higher in healthy donors [15].

### Evidence for regulatory T cell subsets in GBM patients and healthy controls

**Fig. 4:**
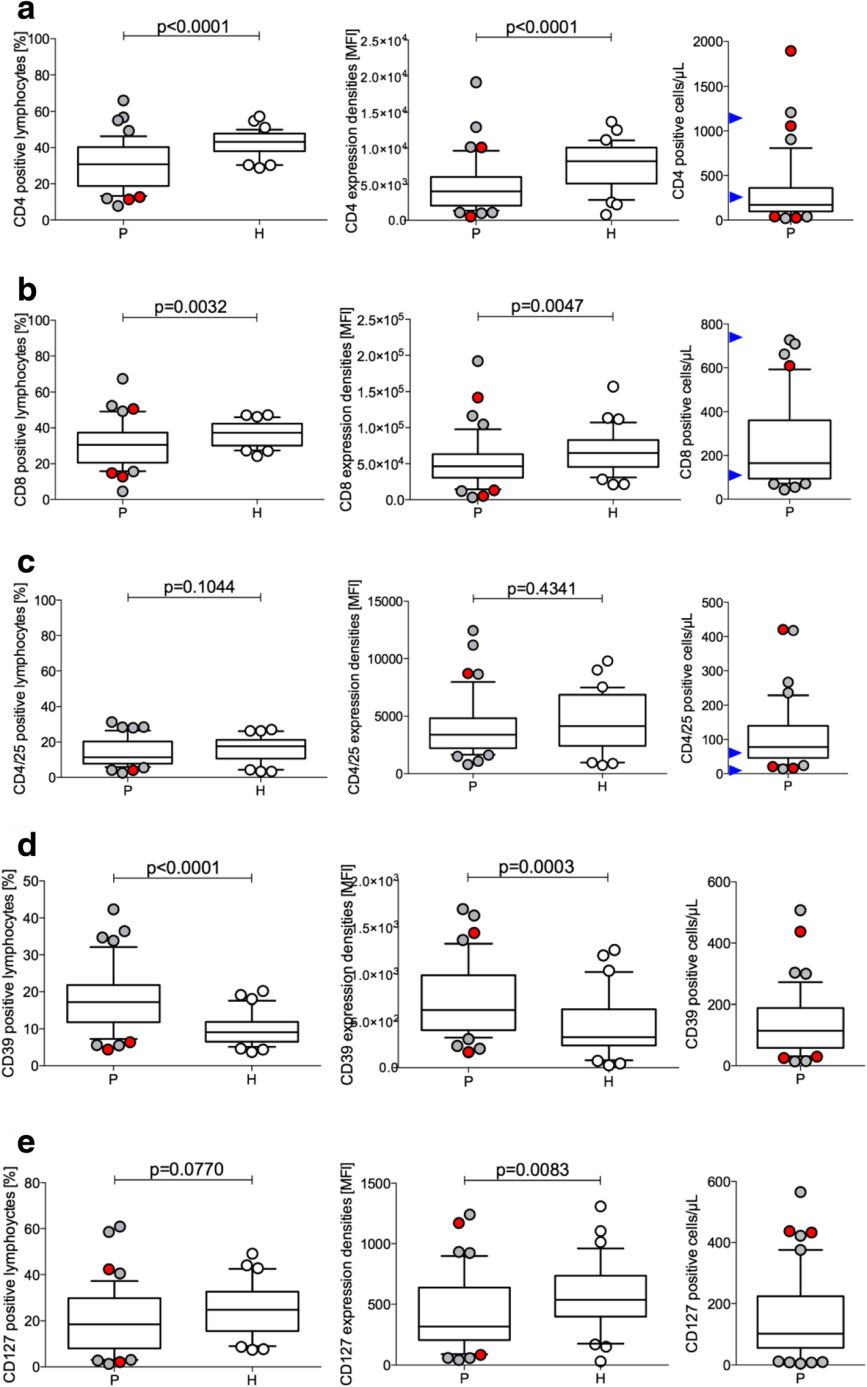
Differences in CD8, CD4, CD4/25, CD39, and CD127-positive lymphocytes. Percent CD cluster-positive lymphocytes are shown for patients (*P*) and healthy donors (*H*) on the left; relative expression densities, given as MFI are shown in the *middle column*, and absolute numbers of CD cluster positive lymphocytes for the patients are shown on the right half of the figure. CD8 (**a**). CD4 (**b**). CD4+/CD25+ (**c**). CD39 (**d**). CD127 (**e**). IDH-1 mutated patients outside of the 25-75 boxed percentile are shown as *red-filled circles*

GBM patients had a very low relative amount of CD4-positive cells (#22, #23, #26, #40) (Fig. 4a), in part corresponding to low absolute numbers. IDH-1-mutated tumor patients display either increased or decreased CD4-positive lymphocytes (Fig. 4b). One IDH-1-mutated patient (#32) had very high CD3-, TCRα/β-, and CD95-positive lymphocytes (Fig. 2). CD8-positive lymphocytes are also heterogeneous between GBM patients and controls (Fig. 4b). This accounts for the percentage of CD8-positive lymphocytes and their expression densities (MFI), as well as the absolute numbers (Fig. 4b). Two patients with high absolute numbers of CD8-positive cells (#24, #28) also presented with high amounts of CD16/CD56-positive cells (Fig. 3b), but not with increased CD3- and TCRα/β-positive lymphocytes (Fig. 2b). The distribution of lymphocytes co-expressing CD4 and CD25 characterizes an activated lymphocyte subpopulation, which contains a large proportion of so-called regulatory, that is, immune suppressive T lymphocytes. The further characterization of CD4/CD25 lymphocytes led to the recognition of the importance of FoxP3 expression, a transcription factor involved in the suppressive function of this cell type. FoxP3 was not determined in our study, but according to a recent review, active FoxP3 is mostly restricted to the CD4/CD25 high T cell subpopulation in humans [17]. According to peripheral blood lymphocyte analyses, neither the median percentage nor the MFI of CD4/CD25-positive lymphocytes in GBM patients and control individuals was significantly different (Fig. 4c). In addition, an important immune suppressive function is displayed by the expression of ATP-degrading enzymes, including CD39; in lymphocytes with low IL-7 receptor, CD127 median values are different between patients and healthy donors (Fig. 4e). Among patients with high absolute numbers of CD39, one IDH-1-mutated patient (#48) presented with very high relative and absolute numbers in addition to high CD39 expression densities (Fig. 4d). Results suggest that CD39 may play a unique role in immune suppression of GBM. Exemplary analysis of GBM tissue preparations suggests that CD39 is also expressed in tumor tissue (Additional file 4: Figure S2). Regulatory T cells (Tregs) are also defined by weak expression of the IL-7 receptor, CD127 (*p* = 0.0083). Figure 4e shows the differences of medians of CD127-positive lymphocytes in GBM and controls as compared with the respective expression densities of CD127. Patients with GBM displayed a lower median of CD127-positive lymphocytes as well as a MFI of CD127. Remarkably, the absolute numbers of CD127-positive cells were also increased in patient #48, who was unique by high CD39 (Fig. 4e).

### Immune phenotypes correlate with survival in GBM

**Fig. 5:**
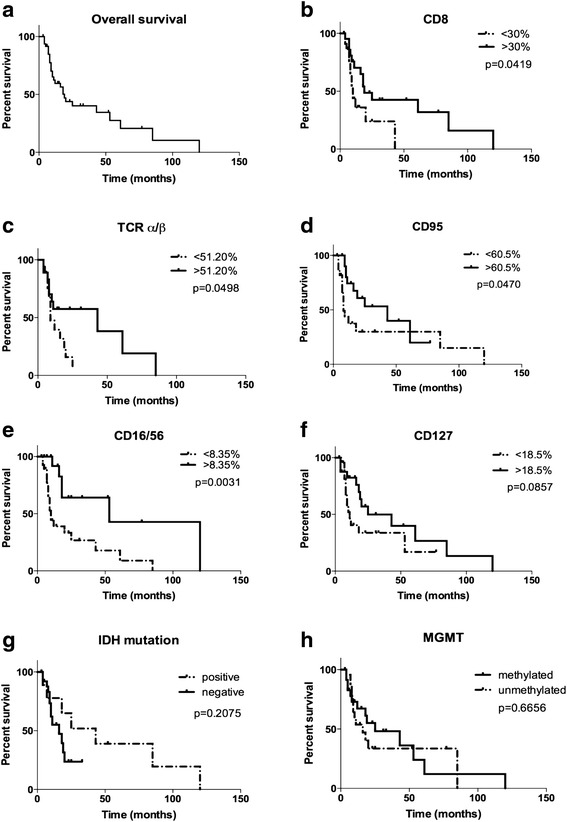
Kaplan-Meier curves of overall survival and lymphocyte subpopulations, IDH-1 and MGMT mutation status. Overall survival of 51 patients with GBM (**a**), distinguished by relative amounts of CD expressing lymphocytes. Patients were grouped according to the median values of markers determined by Mann-Whitney *U* statistical analysis. CD8 cells (**b**), TCRα/β T cells (**c**), CD95 expressing lymphocytes (**d**), CD16/56 activated NK cells (**e**), and CD127 expressing lymphocytes (**f**); IDH-1 mutation status (**g**) and MGMT mutation status (**h**)

Figure 5 shows the overall survival of the GBM patients and the different survival curves in association with immune markers differentially expressed in patients and controls. The median overall survival was 19 months (Fig. 5a). We then analyzed the survival in the context of immune markers. Patients were grouped according to their marker distribution higher or lower of the respective median value. Patients with more than 30 % CD8 expressing differed from those with < 30 % CD8-positive cells, by a better median survival (19 vs. 10 months (*p* = 0.0419), Fig. 5b). TCRα/β expression of >51.2 % as compared to < 51.2 % TCRα/β T cells resulted in significantly better survival of 43 vs. 9 months (*p* = 0.0498; Fig. 5c). CD95 differences were in the same range: With a cutoff of 60.5 %, the median survival was 8 and 43 months, respectively (*p* = 0.047, Fig. 5d). The activation marker CD16 on CD56+ NK cells of (> 8.35 vs. <8.35 %) resulted in a median survival of 53 vs. 10 months (*p* = 0.0031, Fig. 5e). CD127-expressing lymphocytes were analyzed in two groups with > 18.5 vs. < 18.5 % positive cells. The median survival in the low and high CD127 groups was 25 and 11 months, respectively (*p* = 0.0857, Fig. 5f). The IDH-1 mutation showed no evidence for a different survival (*p* = 0.6656, Fig. 5h). Despite strong arguments for lymphocyte subpopulations in GBM patients and survival, high absolute leukocyte counts (c.f. Additional file 5: Figure S3) are related to inferior survival when analyzed by univariable Cox regression analysis and multivariable Cox proportional hazards model (Additional file 2: Table S1, Additional file 3: Table S2, Additional file 6: Figure S4).

## Discussion

In the attempt to answer the question whether the peripheral immune system senses a brain tumor, we investigated lymphocyte subpopulations in peripheral blood shortly before surgery. These analyses are potentially hampered by steroids administered to acutely ill patients before admission. All of our patients had steroid treatment before surgery. However, a major effect on lymphocyte subpopulations was not observed in two patients which were investigated before and 1–3 days after steroid treatment. By contrast, monocyte numbers were significantly affected (Additional file 1: Figure S1).

### GBM patients show reduced amounts of CD3 and TCRα/β

GBM patients presented with reduced numbers and expression densities of CD3 and the TCRα/β. CD3 is an essential member of the TCR complex and different chains of the CD3 complex contribute to signaling of the TCR. T-cell activation results in up-regulation of the CD3 complex. When the CD3-expression density is significantly diminished in patients compared to healthy donors, this indicates impaired responsiveness by TCR-mediated activation. In addition, the expression of the TCRα/β complex was also different between the GBM patients and controls, a fact which may be related to immune suppression by a higher proportion of immune regulatory elements [6] including cytokines, such as IL-10 [18, 19].

### Elevated levels of IL-10 and ferritin in GBM patients

The immune suppressive and activation pathways in GBM were addressed by quantification of two plasma biomarkers: IL-10 and plasma ferritin. The IL-10 median was higher in GBM (*p* = 0.0006). In addition to immune suppression, a direct effect of IL-10 on GBM proliferation has been reported [20, 21]. Higher ferritin was also detected in GBM patients (*p* < 0.0001) and may be related to tumor-associated changes in iron hemostasis [22] as well as macrophage activation, which has also been documented by investigating macrophage infiltration in GBM tissue [23].

### Reduced amounts of CD56 NK cells in GBM patients

Due to low or even negative MHC expression in GBM, the activation of NK cells may contribute to immune surveillance. A difference in NK cell numbers was found in a subpopulation of GBM patients by using the broad NK marker CD56 (Fig. 2). The remaining GBM patients were similar to healthy donors. NK cells may be either cytotoxic (CD56^dim^) or secreting cytokines (CD56^bright^), and co-expressing CD16, when activated [24]. A defined group of our GBM patients (*n* = 16/51) presented with greater numbers of CD56 NK cells (Fig. 3). Since the majority of GBM are low in MHC expression, NK cells have been favored to target GBM tumors by immune therapeutic approaches [25]. Whether activated NK cells detected in a subgroup of GBM patients (Fig. 3b) indeed recognize GBM targets awaits further analysis. Cytokine activation also triggers NK function in CD3-expressing lymphocytes. As shown in Fig. 3c, 5/51 patients and 3/36 healthy donors had increased numbers of such CIK [26]. This cell population can be generated from TCR α/β-positive T cells, acquires NK-receptor expression by cytokine activation, and displays important tumor-specific cytolysis [26]. Absolute numbers of CIK are also higher in four patients, two of which were IDH-1 positive, when compared with reference values of elderly healthy donors [15].

### Reduced amounts of CD4 and CD8 but increased CD39

CIK may also express CD4 and CD8. CD8-positive lymphocytes T cells were distinct in GBM patients both in terms of their relative numbers and expression densities. CD8 is expressed as a homodimer (α/α) in suppressive T lymphocytes presenting with a high CD8 expression density because monoclonal antibodies applied in flow cytometry detect the alpha chain. In contrast, cytotoxic T lymphocytes co-express the CD8β chain and appear with a lower CD8 MFI [27]. Accordingly, GBM patients had higher amounts of potentially cytotoxic, i.e., CD8α/β co-expressing lymphocytes. Absolute numbers of CD8 cells were high in four GBM patients but remained in the normal range of healthy, elderly donors (Fig. 4b). Interestingly, higher absolute numbers of CD8-positive lymphocytes was an important characteristic for better survival, in addition to the IDH-1 mutation status and high KPS according to RPA (Additional file 6: Figure S4). These results are in line with promising results of tumor vaccination attempts [28] and may eventually support vaccination in IDH-1-mutated glioblastoma [14]. We also investigated CD4-positive lymphocyte subpopulations. Similar to a previous study performed by RNA expression analysis [29], we found that GBM patients had less relative amounts of CD4-positive lymphocytes and a reduced expression density of CD4 (Fig. 4a). Absolute amounts of CD4-positive lymphocytes were also lower when compared with reference values of healthy individuals > 50 years of age [30]. An important subpopulation of CD4-positive T cells is the CD4/25 co-expressing activated T cell. Parts of these comprise activated T cells and Tregs. CD4/25-positive Tregs have been previously shown to play a critical role in glioma-related immune surveillance [31]. This observation is supported in appropriate mouse models with experimental brain tumors [32]. In our analysis, the absolute numbers of a CD4/25 population clearly increased in GBM (up to 410/μl, Fig. 4c) when compared with the reference values of CD4/CD25 co-expressing lymphocytes (7–60 cell/μl [30]). In contrast, GBM patients presented with a higher relative amount of another marker protein expressed in Tregs, CD39. CD39 is a membrane-bound nucleotidase, which promotes survival in an ATP-rich environment and counteracts ATP-induced apoptosis [33]. Currently, CD39 positivity is regarded superior to FoxP3 expression in the characterization of Tregs [34, 35]. The median difference of CD39 positivity determined as the percent and expression density (MFI) was significant between the healthy donors and patients (*p* < 0.0001 and *p* = 0.0003, respectively). These observations are in agreement with a previous study analyzing CD39-positive lymphocytes cooperating with CD73 in GBM patients [36]. Preliminary investigation suggests that CD39 expression is positive in GBM tumor tissue (Additional file 4: Figure S2), an observation that needs to be further addressed in the future. Tregs expressing CD39 are further defined by a low CD127 expression density [37, 38]. Indeed, the percent CD127-positive lymphocytes were decreased in GBM patients; results were significant for CD127 fluorescence intensities (Fig. 4e), and some patients were unique by high absolute amounts of CD127 cells (including the IDH-patient #48 with inferior survival).

### Overall survival of GBM patients

**Fig. 6:**
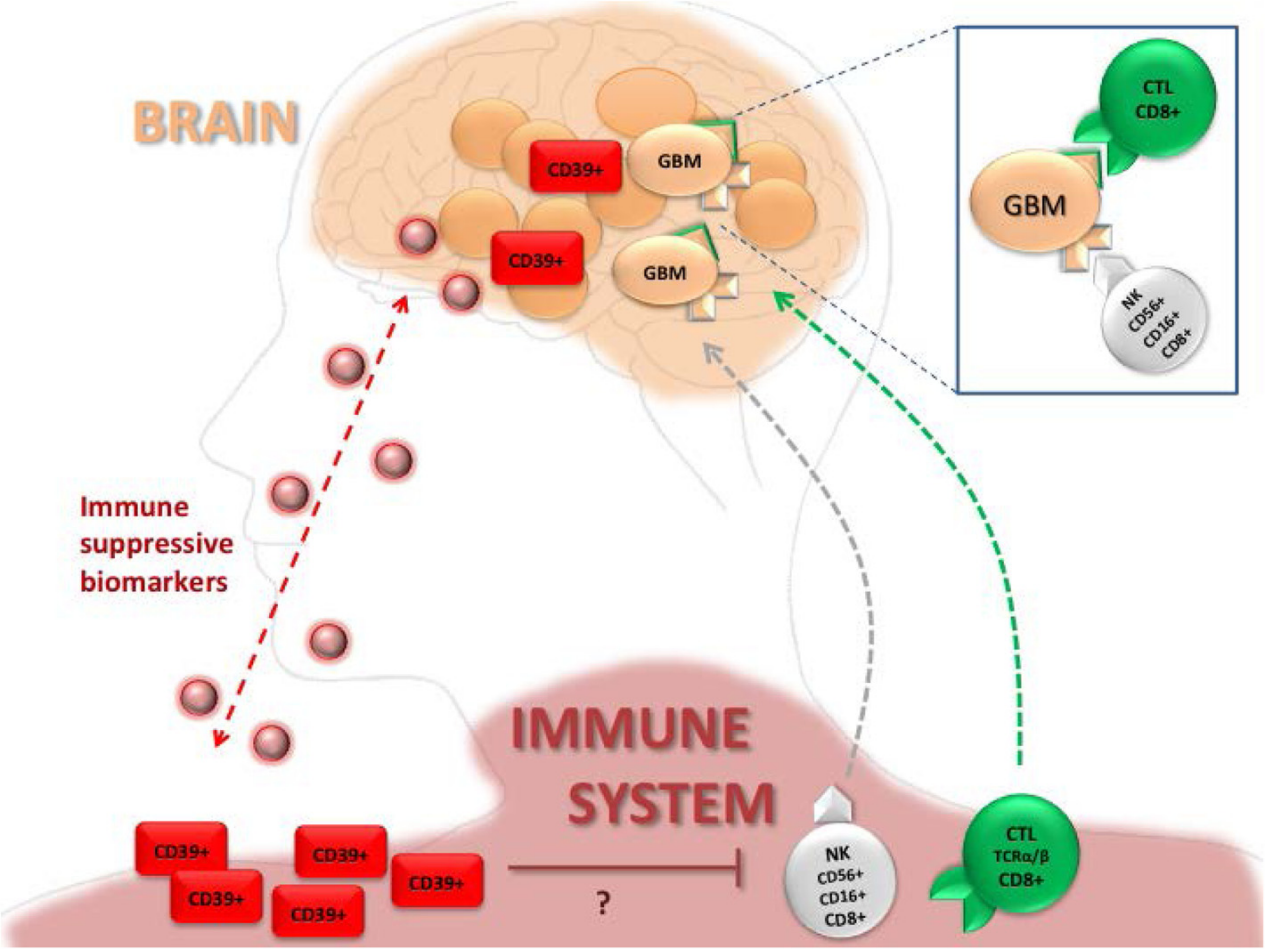
Schematic representation of effector cells guiding immune surveillance in GBM and counter regulatory CD39-positive cells. A number of potential effector cells have been studied for their differential expression in GBM patients at the time of diagnosis. Based on regression analysis of survival data based on the Cox proportional hazards model and model-based recursive partitioning, the beneficial effects of high numbers of CD8-positive lymphocytes and the negative effect by CD39-positive lymphocytes has been demonstrated. Anti-tumor reactive CD8 cells may migrate between the brain and the peripheral tissues. They are likely to be counter regulated by regulatory lymphocytes expressing CD39. CD39 cells may also migrate between tissues or may be induced by immune suppressive biomarkers which can be cytokines but also extracellular vesicles. Tumor tissue is a likely source of such biomarkers.

Kaplan-Meier analysis has been performed for several markers including median of overall survival which was 19 months (Fig. 5), which is similar to published data in patients treated using standard surgery and radio-chemotherapy [39]. In addition to a major influence of neurosurgical intervention [40], immune characteristics of the peripheral immune system may influence tumor recurrence and survival. Expression of TCRα/β and CD8 was related to improved survival (Fig. 5b, Additional file 6: Figure S4); these results correspond to data generated by mRNA profiling [29]. In general, we followed the design of a previous study in which immune markers of blood leukocytes were found to be predictive for survival [10]. Such studies will improve our knowledge about the detrimental cooperative effects between the brain tumor and the peripheral immune system even better when performed as follow-up studies after tumor resection. As a hypothesis provided in Fig. 6, tumor cells appear to induce CD39 expressing regulatory T cells by direct or tumor microenvironment-related interactions. Second, neoantigens expressed by tumor tissues such as mutated IDH-1 may be responsible for the activation of CD8 expressing lymphocytes and a measurable effect on better survival. Third, a better physiology as given by a higher KPS could control the flexibility of the immune system to raise an appropriate immune response. The immune alterations leading to tumor manifestation are possibly related to remodeling of tolerogenic lymphocytes, a process which may be promoted in the elderly [15, 30, 41]. We found a better survival in patients with high relative amounts of CD95 lymphocytes (Fig. 5d). Along these lines, Todo-Bom and colleagues [41] detected more CD95 expression in elderly donors. Activated NK cells may express CD95 and provide direct immune control by recognizing malignant cells with low or negative expression of MHC antigens [42]. Moreover, glioblastoma cells express the CD95 ligand [43] and may be susceptible to CD95 ligand-initiated cell killing and therapy [44]. Moreover, CD95-positive cytotoxic cells may trigger several pathways of caspase activation in glioblastoma cell lines [45]. Although the migration of activated lymphocytes between the peripheral blood and the brain has not been proven, the overall changes of immune phenotypes in GBM patients strongly suggest ongoing crosstalk between the tumor environment and the peripheral immune system (Fig. 6). Based on univariable regression analysis followed by multivariable Cox proportional hazards model and recursive partitioning, the absolute numbers of CD8-positive lymphocytes appear to be relevant for improved survival (Additional file 1: Figure S2). Moreover, the relative amounts of CD39-positive lymphocytes appear to be a negative predictor. The correlation of CD39 (relative amounts) and CD8 (absolute cell numbers) is significant (*p* = 0.0033; *r* = −0.4332, (Additional file 7: Figure S5). A model implementing these variables for survival with functional aspects of immune cells has been provided in Fig. 6. Accordingly, increased amounts of CD8 lymphocytes may eventually invade the tumor tissue and control tumor cell proliferation. Effector cells are likely to be counter regulated by regulatory T cells expressing CD39. In addition to migrating immune cells between the brain and the peripheral immune system, extracellular vesicles released from tumor cells appear to play a major role to potently activate tolerogenic T cells [46, 47], such vesicles may be termed “immune suppressive biomarkers” as well. Further studies and follow-up may substantiate the current working hypothesis. After all, immunogenicity remains to be the most important factor to control malignancies. This hypothesis is currently supported by a likely successful approach to immunize IDH-1 mutation-positive tumor patients with IDH-1-derived peptides [14] and improved survival of IDH-1-mutated patients [48].

## Conclusions

Immune phenotypes in individual patients with GBM indicate sensitization as well as anergy against glioblastoma tumor tissue. These observations may be exploited to individualized immune-based therapeutic interventions to eventually improve survival. Parameters can be also used for clinical studies. Most important predictors are mutated genes such as IDH-1, a patient's KPS, the ATP nucleotidase CD39, and CD8 positive cytotoxic cells.

## Additional files

Additional file 1: Figure S1. GBM patients before and after steroid administration. (DOCX 690 kb) Additional file 2: Table S1. Univariable Cox regression analysis. (DOCX 15 kb) Additional file 3: Table S2. Multivariable proportional hazards model. (DOCX 25 kb) Additional file 4: Figure S2. CD39 expression in GBM tumor lysate. (DOCX 206 kb) Additional file 5: Figure S3. Absolute numbers of leukocytes. (DOCX 163 kb) Additional file 6: Figure S4. Recursive Partitioning Analysis (RPA). (DOCX 94.7 kb) Additional file 7: Figure S5 Spearman rank correlation of CD8 and CD39. (DOCX 183 kb)

## Abbreviations

CD: cluster of differentiation
CIK: cytokine-induced killer(s)
GBM: glioblastoma multiforme
IDH-1: isocitrate dehydrogenase-1
IL: interleukin(s)
MFI: mean fluorescence intensity
MGMT: O^6^-Methylguanine-DNA methyltransferase
MHC: major histocompatibility complex
NK: natural killer
RPA: recursive partitioning analysis
TCR: T cell receptor complex
Treg: regulatory T cell(s)
