# Supplementary material for: Immune phenotypes predict survival in patients with glioblastoma multiforme
Source: J Hematol Oncol. 2016 Sep 1;9(1):77. doi: 10.1186/s13045-016-0272-3 (PMC5009501; doi:10.1186/s13045-016-0272-3)
Supplement: Additional file 1: Figure S1. — GBM patients before and after steroid administration. (DOCX 690 kb) [file 13045_2016_272_MOESM1_ESM.docx]

**Supplementary Figure S1: GBM patients before and after steroid administration**

Changes in lymphocyte subpopulations by high dose steroid treatment in 2 patients with GBM.


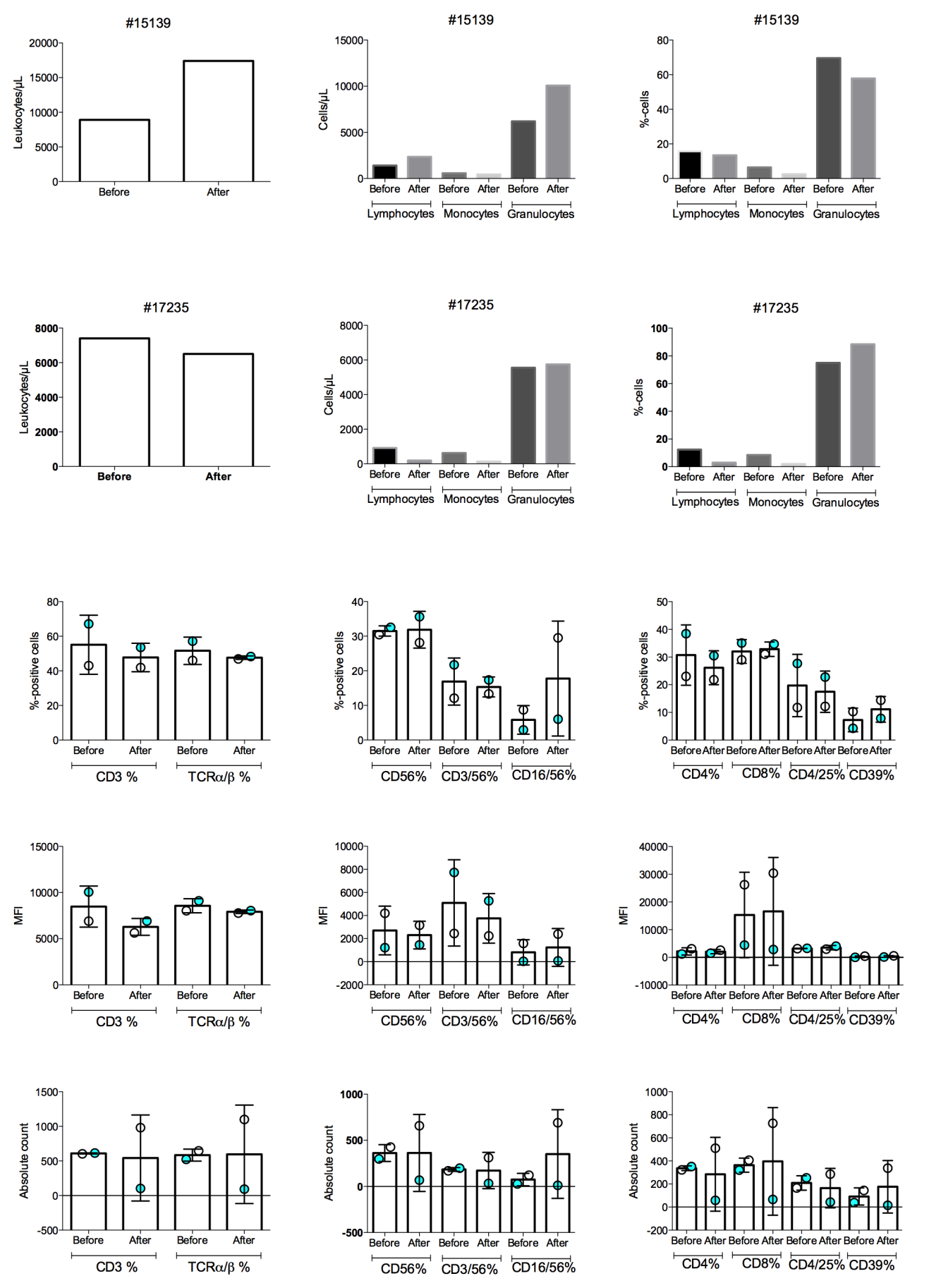


According to flow cytometric analyses, monocytes were significantly diminished after 24-48h following steroid medication. The changes differed in the two patients analyzed. Both patients had an elevation of CRP after steroid medication. The relative amounts of lymphocyte subpopulations were very constant before and after steroids. The absolute numbers changed more.

Fig. S1.

EDTA-anti-coagulated blood samples were subjected to flow cytometric analysis. Percent positive lymphocytes and absolute cell counts were determined by Cellquest software (BD Biosciences.com) and routine differential blood count analysis. Open circles (#15139) and blue-filled circles (#17235) represent individual patients. One patient also had an elevation of CRP (C-reactive protein) after steroid medication indicating some signs of an infection.
