# Supplementary material for: Immune phenotypes predict survival in patients with glioblastoma multiforme
Source: J Hematol Oncol. 2016 Sep 1;9(1):77. doi: 10.1186/s13045-016-0272-3 (PMC5009501; doi:10.1186/s13045-016-0272-3)
Supplement: Additional file 2: Table S1. — Univariable Cox regression analysis. (DOCX 15 kb) [file 13045_2016_272_MOESM2_ESM.docx]

**Supplementary Table S1: Univariable Cox regression analysis**

P-values of univariable Cox regression analysis to pre-select biomarkers for a multivariable proportional hazards model on overall survival using the criterion p < 0.2 (n=51).

| **Parameters*** | **p-value** |
| --- | --- |
| Leukocyte count | 0.125 |
| Granulocyte count | 0.045 |
| CD3 | 0.093 |
| TCRα/β | 0.089 |
| CD8 | 0.034 |
| CD127 | 0.165 |
| CD95 | 0.078 |
| CD16/56 | 0.181 |
| Lymphocyte Count | 0.182 |
| Monocyte Count | 0.457 |
| CD39 | 0.270 |
| CD56 | 0.356 |
| CD3/56 | 0.677 |
| CD4 | 0.287 |
| CD4/25 | 0.770 |

*absolute numbers were included for the analysis

Parameters which showed a p-value <0.2 in univariable analysis were included for the multivariable Cox proportional Hazard model. Only absolute numbers of leukocytes with the marker profile indicated gave relevant p-values, whereas relative numbers (% positives) and MFI (mean fluorescence intensities) did not. The analysis of absolute numbers included all immune markers shown in Figs. 1-5, leukocyte counts, KPS category (Karnofsky Performance status Scale), MGMT-Status, IDH--mutation status, and extent of surgical resection. The strongest predictors of longer survival based on the Hazard ratio (HR) were high absolute numbers of granulocyte counts, high amounts of TCRα/β positive T cells and CD8-positive T cells, low absolute leukocyte counts and low absolute numbers of CD95-positive lymphocytes, as well as a the positive IDH-1 mutation status and high KPS categories.
