# Supplementary material for: Immune phenotypes predict survival in patients with glioblastoma multiforme
Source: J Hematol Oncol. 2016 Sep 1;9(1):77. doi: 10.1186/s13045-016-0272-3 (PMC5009501; doi:10.1186/s13045-016-0272-3)
Supplement: Additional file 3: Table S2. — Multivariable proportional hazards model. (DOCX 25 kb) [file 13045_2016_272_MOESM3_ESM.docx]

**Supplementary Table S2: Multivariable proportional hazards model**

Results of a multivariable proportional hazards model on overall survival (final model after backward selection from the set of all parameters with p < 0.2; n=51). *absolute numbers were included for the analysis. The hazard ratio is given per unit (cells per microliter blood) with the exception of IDH-1-mutation status (where the reference class is "negative", corresponding to wild type IDH-1).

HR, hazard ratio, CI, confidence interval

| **Parameters*** | **p** | **HR** | **95% CI for HR** | |
| --- | --- | --- | --- | --- |
|  |  |  | **Lower** | **Upper** |
| Leukocyte count* | 0.0028 | **2.991** | 1.458 | 6.139 |
| Granulocyte count* | 0.0036 | 0.999 | 0.998 | 1.000 |
| TCRα/β* | 0.0100 | 0.995 | 0.991 | 0.999 |
| CD8* | 0.0401 | 0.994 | 0.988 | 1.000 |
| CD95* | 0.0488 | 1.004 | 1.000 | 1.008 |
| IDH-1 status^$^ | 0.0036 | 5.093 | 1.700 | 15.258 |
| KPS | 0.0108 | 0.962 | 0.933 | 0.991 |

*absolute cell numbers were included for the analysis

^$^IGH-1 mutation status according to immune pathological results^1^

KPS: Karnofsky Performance status Scale

1. Capper D, Weissert S, Balss J, Habel A, Meyer J, Jager D, Ackermann U, Tessmer C, Korshunov A, Zentgraf H, Hartmann C, von Deimling A: Characterization of R132H mutation-specific IDH1 antibody binding in brain tumors, Brain Pathol 20:245-254
