# Supplementary material for: Immune phenotypes predict survival in patients with glioblastoma multiforme
Source: J Hematol Oncol. 2016 Sep 1;9(1):77. doi: 10.1186/s13045-016-0272-3 (PMC5009501; doi:10.1186/s13045-016-0272-3)
Supplement: Additional file 4: Figure S2. — CD39 expression in GBM tumor lysate. (DOCX 206 kb) [file 13045_2016_272_MOESM4_ESM.docx]

**
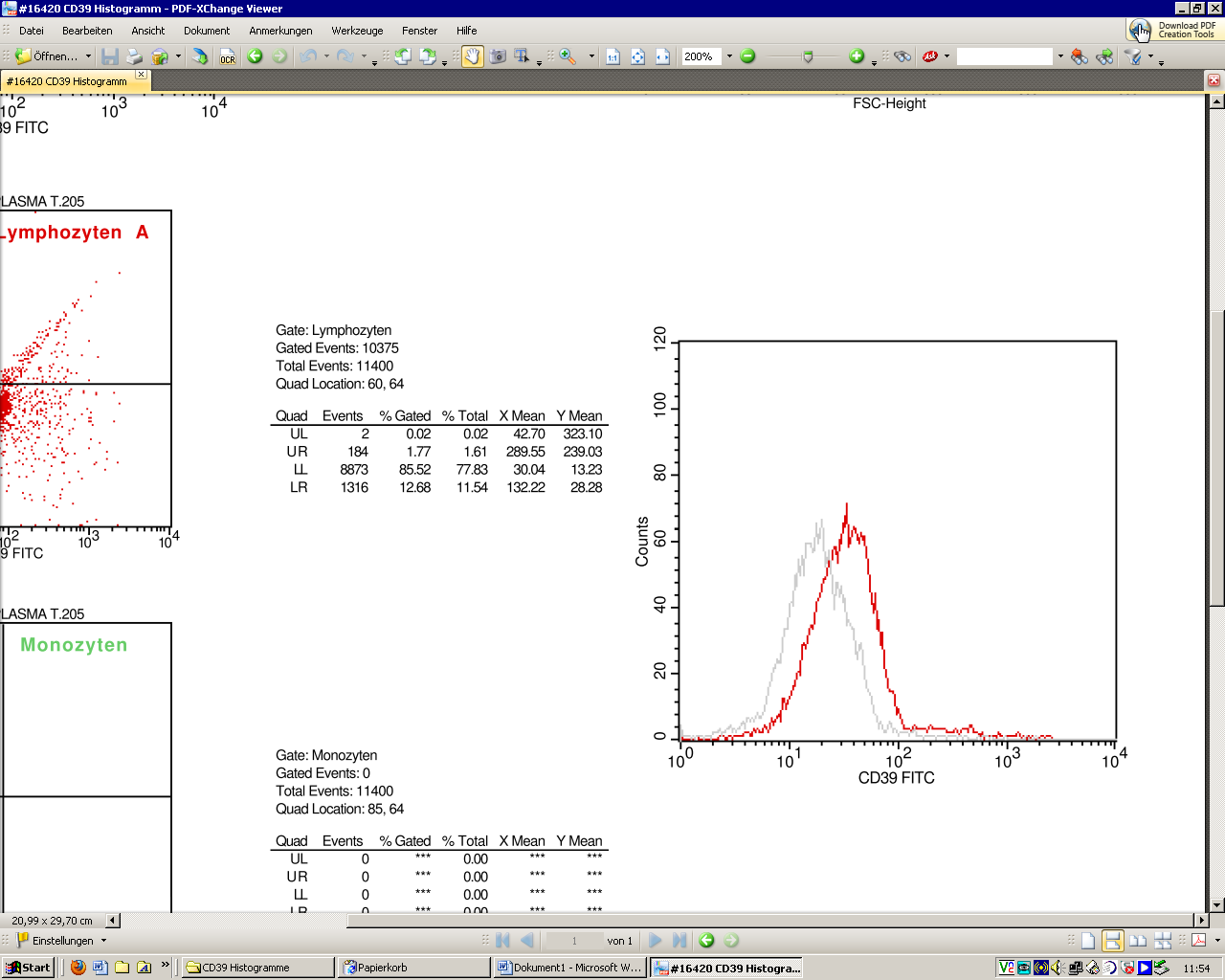
Supplementary Figure S2: CD39 expression in GBM tumor lysate**

Flow cytometric analysis of CD39 positive cells in fresh GBM tumor lysate.

Fig. S2.

Flow cytometric analysis of CD39 expression in a fresh tumor isolate. Fresh tumor material was mechanically dissected, and then trypsinized (2.5% trypsin, MerckBiochrom.com) for 2.5h, and single cells were enriched by ficoll separation. Cells were fixed and permeabilized using Perm/Fix (BD Biosciences.com) and washed with Perm/Wash (BD Biosciences.com) and blocked with normal rabbit IgG (DAKO), followed by staining with anti-CD39-FITC (clone A1; Serotec.com) and examined by flow cytometry using FACScalibur and CellQuestTM software (BD Biosciences.com). Grey line (negative control; red line CD39 stained glioblastoma tumor cells).
