# Supplementary material for: Immune phenotypes predict survival in patients with glioblastoma multiforme
Source: J Hematol Oncol. 2016 Sep 1;9(1):77. doi: 10.1186/s13045-016-0272-3 (PMC5009501; doi:10.1186/s13045-016-0272-3)
Supplement: Additional file 5: Figure S3. — Absolute numbers of leukocytes. (DOCX 163 kb) [file 13045_2016_272_MOESM5_ESM.docx]

**Supplementary Figure S3: Absolute numbers of leukocytes**

Distribution of absolute numbers of leukocytes and leukocyte subpopulations in GBM patients

| Absolute leukocyte counts  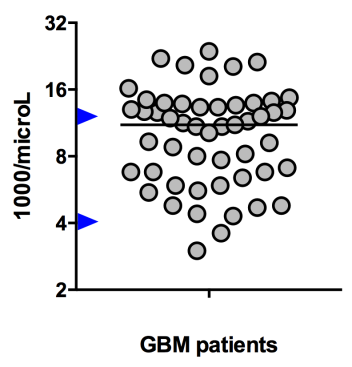 | Absolute lymphocyte, monocyte and granulocyte counts  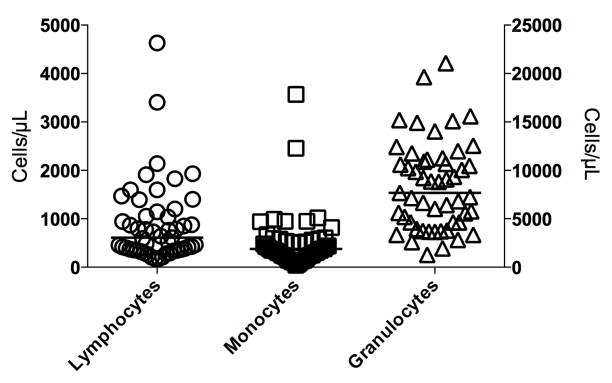 |
| --- | --- |
| Distribution of leukocyte counts in patients with GBM before surgery. Range of age-matched donors is given by blue arrow heads on the y-axis. | Distribution of absolute lymphocyte, monocyte and granulocyte counts of patients with GBM before surgery. |
