# Supplementary material for: Immune phenotypes predict survival in patients with glioblastoma multiforme
Source: J Hematol Oncol. 2016 Sep 1;9(1):77. doi: 10.1186/s13045-016-0272-3 (PMC5009501; doi:10.1186/s13045-016-0272-3)
Supplement: Additional file 6: Figure S4. — Recursive Partitioning Analysis (RPA). (DOCX 94.7 kb) [file 13045_2016_272_MOESM6_ESM.docx]

**Supplementary Figure S4:**

**Recursive Partitioning Analysis**

Recursive partitioning analysis1 with respect to overall survival based on sex, age, Karnofsky Performance status Scale (KPS), IDH-1- and MGMT- mutation status, absolute leukocyte counts, absolute and relative amounts of granulocytes, lymphocytes, monocytes as well as all immune markers was performed. KPS, IDH_1, CD8_abs (absolute counts in blood samples), KPS, IDH_1 (mutation status), CD39_r (%, relative amounts of blood lymphocytes) were identified as splitting variables which differentiate between risk classes for overall survival. Corresponding cut off values is given. The leftmost figure is based on absolute counts, the central figure on MFI, the rightmost figure on relative counts.

| **RPA Analysis based on absolute amounts** | **RPA Analysis on MFI** | **RPA Analysis based on relative amounts of cells** |
| --- | --- | --- |
| 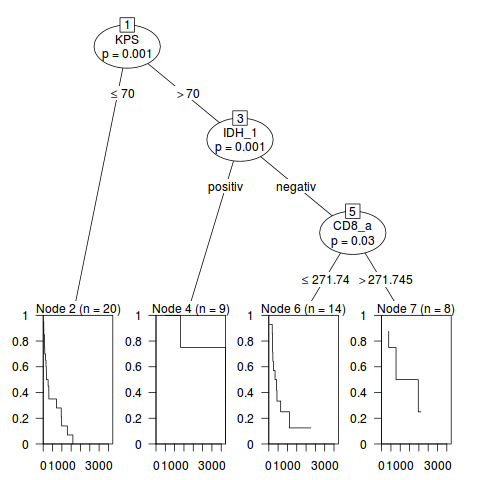 | 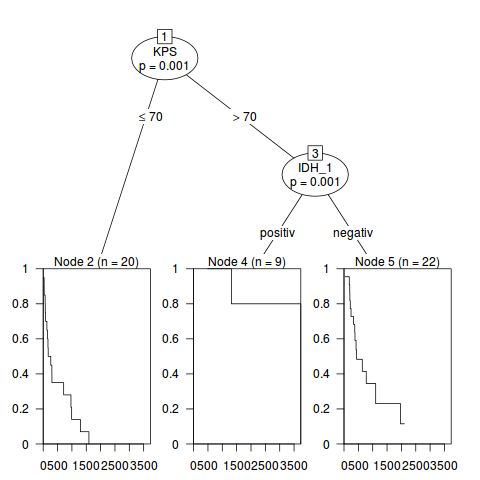 | 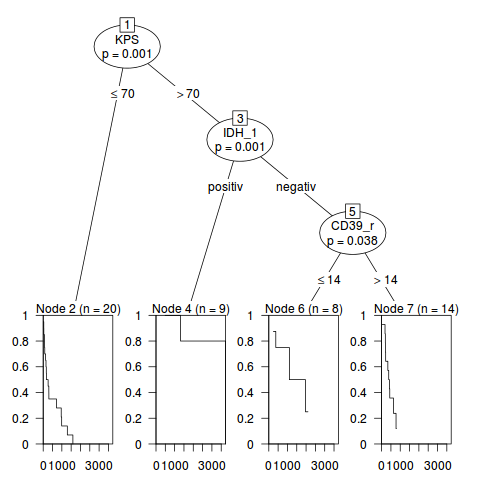 |
| KPS, IDH_1 and absolute counts of CD8 positive lymphocytes had an impact on survival, cut-off value for KPS was 70 and for CD8 within IDH_1 negative patients 271.74 cells/µl. | In patients with KPS >70, IDH-1 mutated patients had a better outcome than IDH-1 wild type patients | CD39 is an important antigen expressed by regulatory T lymphocytes. Among all immune markers analyzed, relative amounts of CD39-positive lymphocytes (cut-off value: 14%) were linked to impaired survival. at least in a subgroup of patients. |

Ref.

Hothorn T, Hornik K, Zeileis A (2006). “Unbiased Recursive Partitioning: A Conditional Inference Framework.”Journal of Computational and Graphical Statistics, 15(3), 651–674.
