# Supplementary material for: Immune phenotypes predict survival in patients with glioblastoma multiforme
Source: J Hematol Oncol. 2016 Sep 1;9(1):77. doi: 10.1186/s13045-016-0272-3 (PMC5009501; doi:10.1186/s13045-016-0272-3)
Supplement: Additional file 7: Figure S5 — Spearman rank correlation of CD8 and CD39. (DOCX 183 kb) [file 13045_2016_272_MOESM7_ESM.docx]

**Supplementary Figure S5: Correlation of CD8 and CD39**

Spearman rank correlation coefficient of absolute numbers of CD8 positive lymphocytes and relative amounts of CD39-positive lymphocytes in GBM


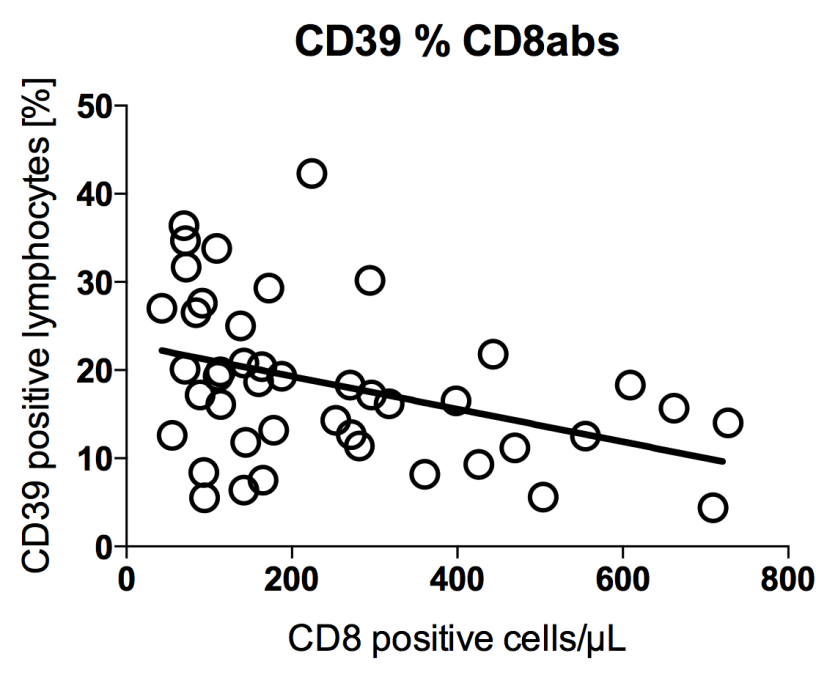


Fig. S5.

Linear regression analysis of absolute numbers of CD8-positive cells and percent CD39 positive lymphocytes in peripheral blood samples of GBM patients are negatively correlated (R= -0.4332 (p= 0.0033)

(numbers of x/y pairs=44; calculated by GraphPadPrism vs. 6.0).
